# Development and psychometric testing of the Visual Analogue Scale for Irritable Bowel Syndrome (VAS-IBS)

**DOI:** 10.1186/1471-230X-7-16

**Published:** 2007-05-03

**Authors:** Mariette Bengtsson, Bodil Ohlsson, Kerstin Ulander

**Affiliations:** 1 Division of Gastroenterology and Hepatology, Department of Medicine, Malmö University Hospital, Sweden; 2 Department of Health Sciences, Faculty of Medicine, Lund University, Sweden; 3 Department of Clinical Sciences, Faculty of Medicine, Lund University, Sweden; 4 Department of Health Sciences, Kristianstad University, Sweden

## Abstract

**Background:**

The aim of this study was to develop and psychometrically test a short, patient-reported questionnaire to be used in clinical practice for patients with Irritable Bowel Syndrome (IBS). The Visual Analogue Scale for Irritable Bowel Syndrome (VAS-IBS) questionnaire was designed to measure the treatment response of symptoms and well-being in patients suffering from IBS.

**Methods:**

The VAS-IBS was psychometrically tested for content and criterion validity, scale acceptability, item-reduction, internal reliability consistency, simplicity, and speed. Two samples were used. One expert panel (five physicians and four registered nurses), who gave their opinion on the content validity, and one of 71 patients with IBS (mean age 38 years SD +13, range 19–65), who completed the VAS-IBS, as well as the Gastrointestinal Symptom Rating Scale and the Psychological General Well-Being Index for criterion validity.

**Results:**

The items in the VAS-IBS capture the main physical concerns women with IBS might present and the psychometric testing confirmed that the VAS-IBS is an acceptable homogeneous patient-reported questionnaire indicated by Cronbach's alpha internal consistency reliability coefficient, with a value of 0.85. All correlations to test the criterion validity performed by using Pearson's correlation test, were statistically significant (p < 0.0001) and in the expected directions. The VAS-IBS is easy to complete and unproblematic to calculate.

**Conclusion:**

The VAS-IBS appears to be reliable and user-friendly, for patients as well as for health professionals. The final version of the VAS-IBS including nine items needs to be further tested in clinical practice cross-culturally in women as well as in men.

## Background

Irritable Bowel Syndrome (IBS) is a common, global, functional, gastrointestinal disorder, affecting a significant number of people, predominantly women [1,2]. Etiology and pathophysiology is insufficiently understood, but it is generally accepted that the symptoms of IBS are multidetermined, and can be explained as a biopsychosocial model [3]. Early life factors can later in life influence the patient's psychosocial experience and physiological function, and generate gastrointestinal symptoms in a vulnerable patient. The inter-relationship between the patient's psychosocial status and physiology will affect how the patient experiences the symptoms, the patient's reaction, as well as the clinical outcome [3]. IBS has traditionally been considered as a diagnosis of exclusion rather than a primary diagnosis, since there are no observable biochemical and/or structural abnormalities to be found [4,5]. The diagnosis IBS is based on the Rome criteria and a third version has been presented during spring 2006 [3,6] (Table 1). Abdominal pain and bloating are the dominant symptoms of IBS [7-9], and also the most troublesome [8,10].

It is difficult in clinical practice to estimate the symptomatic changes occurring in patients having IBS based on to their description, as well as comparing the effect of different treatments. There is a need to translate the patients' perception of their symptoms and their subjective well-being into numbers. A patient-reported, short (less than 10 items) and reliable measuring questionnaire could be of help. It is important that the questionnaire is straightforward to calculate and easy to understand for patients as well as for health professionals, to maintain a high adherence from both groups. This questionnaire should serve as a complement to the anamnesis and measure the response to treatment of symptoms related to IBS as well as the general well-being of the patient. A search in Medline and Cinahl in 2001 did not show the existence of a questionnaire that fulfilled these criteria. The identified questionnaires showed reasonable psychometric and methodological qualities [11-13], but not one of the questionnaires identified is optimal in all aspects [13]. The identified questionnaires have too many (more than 10) or inadequate questions (often only focus on pain) to assess outcomes of interventions in daily clinical practise, and are therefore not suitable to be used.

The aim of this study was therefore to develop and psychometrically test a short, patient-reported questionnaire to be used in clinical practice by different health care professionals to assess the status over time for patients with established IBS.

## Methods

### The development of the Visual Analogue Scale for Irritable Bowel Syndrome (VAS-IBS)

#### Creating suitable items

Items to be used in a questionnaire can be generated from the literature [14,15], and to get a conceptualization of the construct of interest a literature review of symptoms and inconveniences experienced by patients with IBS was performed. The most outstanding physical symptoms identified were divided into six main groups; *Abdominal Pain, Diarrhoea, Constipation, Bloating and Flatulence, Abnormal bowel passage *and *Vomiting and Nausea*. All symptoms except *Vomiting and Nausea *also support the diagnosis of IBS. Symptoms included in the group *Abnormal bowel passage *(straining, urgency or feeling of incomplete evacuation) might also be connected to diarrhoea, to constipation as well as to the use of laxatives [16] and these inconveniences have been reported by patients as minor problems [7]. Since it might be difficult to evaluate symptoms associated with *Abnormal bowel passage *on a scale, and the VAS-IBS should serve as a complement to the anamnesis, it may be preferable to discuss these symptoms. One item for each physical symptom was created; *Abdominal Pain, Diarrhoea, Constipation, Bloating and Flatulence*, and *Vomiting and Nausea*. An overall item concerning the patient's bowel symptoms was also included in the questionnaire, and the intension was to use it as a control item for psychometric testing. This item was supposed to be removed in the final version of the VAS-IBS.

Besides physical health problems, IBS also has a negative influence on a person's mental health [17,18] as well as on his/hers daily life [19,20], and therefore questions related to these subjects were added. However, the focus of the questionnaire should still be on the patient's physical symptoms. Totally three items were created. One item related to mental health and two items related to daily life to see which of them best corresponded to the intention of the questionnaire. It is a common approach to have a number of items addressing a single underlying characteristic, and to use only one, all-inclusive, self-administered, single, global and item-specific question of each concept may appear to be insufficient. However, a single question may well provide information on all aspects of a phenomenon, and a summary of an individual's perception [21].

The time window was set to *during the previous week*, since that is the same time window as for the Gastrointestinal Symptom Rating Scale (GSRS) and the Psychological General Well-Being Index (PGWB), which were chosen as comparable questionnaires to test the criterion validity of the VAS-IBS.

#### The method of the scale

The purpose of the VAS-IBS was to form a clinical an opinion of each of the patient's health related complaints over time, and not to calculate a final score. The visual analogue scale (VAS) was chosen for each of the items. The method of VAS has earlier been used to measure well-being and symptoms in patients with IBS [22-25]. The VAS is preferable to graded scales, since the steps between the descriptive terms are not known [26]. If a graded scale contains words, one can not be sure that the true feeling of the respondent has been captured, since a respondent's view of the meaning of a word may not correspond to the researcher's view [27]. Visual analogue scale can be used regardless of language [28], which is an advantage since health professionals meet patients in clinical practice from different cultures. The VAS has been shown to be a reliable scale with results easily calculated [26,29,30]. However, a change of one point or more on a 7-point Likert scale might be easier to pinpoint for the health professionals, than a change of 10-points on a VAS [29]. On the other hand, the patient has more of a choice on a VAS. In the VAS-IBS the patients were supposed to record the overall severity of each item on a 100 millimetre long line (very severe discomfort = 0 to no discomfort at all = 100) [15]. Horizontal lines seem to be preferred to vertical lines [31]. Graduation, verbal or numerical labels were not placed along the line, as there was a risk that there might be a clustering of responses beside the labels [30], and thereby make the results more difficult to judge.

### Samples

Two separate samples were used, one expert panel as well as patients suffering from IBS. The members of the expert panel were chosen by the author and a physician. The panel consisted of doctors and nurses who meet patients with IBS in daily clinical practice, two professors and three physicians all specialized in gastroenterology, and five registered nurses. Their assignment was to give their opinion on the content validity of the VAS-IBS by the use of a content validity index [32]. The questionnaire, together with an information letter was mailed to the members of the expert panel in November 2004. A reminder was sent out to a non-responding member (a registered nurse), but there was no reply. In all, the responses of nine members were analyzed.

To identify suitable patients who had visited a gastroenterology clinic for long and short time as well as only a primary care centre, an enquiry was made among patients who had visited a gastroenterology university clinic in Sweden, between 1 January 1998 and 20 November 2005 and among those who had been referred for a second opinion to the same clinic between 15 May 2003 and 15 May 2004. The referred patients were recruited before they visited any health care professional working at the hospital. Patients with severe diseases, for example liver or kidney diseases, chronic pulmonary or heart diseases, were excluded. Only patients with IBS with symptoms present in the previous month were qualified to participate. No more than five men were identified and they were not included, since they were few and there are differences between women and men concerning symptoms related to IBS [10,33]. Extra intestinal symptoms such as headache and other chronic pain are also more common in women [34] and they report a lower Health-Related Quality of Life [10,35,36].

In total, 181 female patients who fulfilled the Rome II criteria for IBS [37] verified by the patients' medical record, were identified and invited to participate in research, but 110 did not want to participate in any offered study. Sixty-eight women did not reply to our request, and the reasons the other 42 women gave for refraining were "not interested" (*n *= 25), "no symptoms at the moment" (*n *= 6), they did not complete any of the questionnaires (*n *= 6) or "wanted to participate but could not come" (*n *= 5). In total 71 patients (mean age 38 years SD ± 13, range 19–65) with a duration of IBS for 2 to 50 years were included. All patients taken part in this process were at the time of recruitment offered to take part in two research projects, (LU-510-02 and LU 735-02), approved by the local Ethics Committee of Lund University. They all reviewed and signed a written, informed consent, and completed the three questionnaires; the VAS-IBS, the GSRS and the PGWB. All questionnaires were sent to the patients by mail, together with written information. The questionnaires were collected at the hospital when they were signed up for one of the two research projects.

### Questionnaires

The content validity index consisted of instructions and 31 questions about the VAS-IBS. Each expert panel member graded the content relevance, agreement of the definition of the item, and the relevance of the scale for each of the nine items in the VAS-IBS [38], on a four point scale (4 reflects total relevance and 1 indicates total irrelevance). The experts were also asked to give an opinion on the application of the VAS-IBS, guided by four questions in the end of the questionnaire. The content validity index has been validated by experts in the field and evaluated for face validity.

The GSRS is a Swedish disease-specific questionnaire designed to evaluate gastrointestinal symptoms. It was originally constructed as an interview-based rating scale [39], and was later modified to become a self-administered questionnaire [40,41]. The questionnaire includes 15 items divided into five dimensions; Abdominal Pain Syndrome (3 items), Reflux Syndrome (2 items), Indigestion Syndrome (4 items), Diarrhoea Syndrome (3 items), and Constipation Syndrome (3 items). Each item is evaluated on a seven-grade Likert scale and gives a total range-value between 15 and 105, i.e. the higher the scores, the more pronounced are the symptoms.

The PGWB is a generic questionnaire to be used to measure positive and negative aspects of psychological well-being and distress [42]. The questionnaire includes 22 items divided into six dimensions: Anxiety (5 items), Depressed mood (3 items), Positive well-being (4 items), Self-control (3 items), General health (3 items) and Vitality (4 items). A six-grade Likert scale is used to measure the items, which gives a total range-value between 22 and 132, i.e. the higher the value, the better is the patient's psychological well-being.

The GSRS and the PGWB have been used on Swedish patients with functional bowel disorders [36,43], and norm values for healthy controls have been described [35].

### Psychometric characteristics

VAS-IBS was psychometrically tested to study whether it measured the construct of interest, and how the questionnaire behaved in relation to a variety of conditions. All statistical analyses were carried out by SPSS 11.0 for Windows^®^. A *p *value <0.05 was considered statistically significant.

#### Content validity

There are no statistical tests or other objective methods available to prove how adequately the sample of questions reflects the purpose of a measurement, but face validity considered by one or several experts can be used [15,38]. The content validity of the VAS-IBS was the methodological judgement made by experts in the content area, and they completed a content validity index. The level of agreement was set to no more than one expert panel member scoring an item less than 3 on a four-point scale [44].

#### Criterion validity

The concurrent validity was the approach chosen to establish the criterion validity of the VAS-IBS. It was measured by a calculation of the relation between the items related to physical symptoms in the VAS-IBS and the GSRS as well as between the item concerning mental health in the VAS-IBS and the total sum of the PGWB. The item concerning *Abdominal Pain *was compared to the dimension Abdominal Pain (GSRS), the item concerning *Diarrhoea *to the dimension Diarrhoea (GSRS), the item concerning *Constipation *to the dimension Constipation (GSRS), and the item concerning *Bloating and Flatulence *to the dimension Indigestion (GSRS). The item concerning *Vomiting and Nausea *was compared to only one of the two items in the dimension Reflux (GSRS), since the other item was about heartburn. The relationships were described by calculating a correlation coefficient by the Pearson's correlation test. A correlation coefficient range from +1.00 for a perfect and positive correlation to 0.0 for no correlation at all, to -1.00 for a perfect negative correlation [15].

#### Item reduction

Before an item was removed on the basis of the comments of the expert panel, the clinical importance and relevance of the item were judged. Pearson's correlation test was used to assess the correlation between all the items in the VAS-IBS [15,45]. In present study items that had a high correlation (>0.80) were considered for removal, as well as items that had >5 percent missing data.

#### Test-retest reliability

The test-retest reliability was performed on the first version of the VAS-IBS, and a reasonable intra-class correlation coefficient value is according to Streiner and Norman [15] about 0.70. Expert opinions regarding the appropriate interval vary from an hour to a year depending of the situation, but two weeks are normal [15]. In this study the time interval was set to four weeks, before the patients were included in other in other research projects.

#### Acceptability of the scale

Concerning the relevance of the scale, the comments of the expert panel were taken into account. The scale score distribution was considered from four aspects; *score span, mean score, scale floor and ceiling effects, and scale skewness*. The scale was considered acceptable when observed scores were well distributed and the mean scores were near the midpoint of the scale [14]. There are no generally accepted criteria for floor and ceiling effects and the limits in this study was set at 20 percent or less [14]. The skewness statistics were acceptable between -1 and + 1 [14].

#### Internal consistency reliability

Taken into account that IBS can be explained as a biopsychosocial model [3], the VAS-IBS should be regarded as a one-dimensional questionnaire, despite that the items included are related to several health aspects. Alpha-if-item deleted was used to assess the degree of consistency or homogeneity of the items in the final version of the VAS-IBS, after item reduction. The higher the value, the more reliable is a measuring instrument. A minimum coefficient of 0.70 was required for the Cronbach's alpha internal consistency reliability coefficient for the items, but not higher than 0.90 [15,45].

#### Other aspects

The simplicity and the speed with which the VAS-IBS was completed were also taken into consideration as well as number of internal missing data.

## Results

### Content validity

The expert panel agreed that the VAS-IBS gives a good picture of how a patient with IBS might feel, and all members except one thought that the VAS-IBS might be useful in clinical practice. Agreement was achieved for all items concerning the relevance, as well as the definitions of all items. One item (quality of life) achieved total consensus concerning its relevance (all experts scored 4). The results of the experts' agreement can be seen in Table 2. However, some members of the panel suggested the inclusion of additional items. One expert suggested that items related to the *sensation of incomplete evacuation *and the *urgency to defecation *should be added. Another expert suggested adding an item related to *the patient's daily life *and a third suggested that an item about *food intolerance *might be of value. One expert suggested that the patient's definition of each symptom should be registered in the questionnaire, but did not give any advice on how this should be done.

### Criterion validity

The calculations were performed by using Pearson's correlation test, and all 71 completed VAS-IBS questionnaires were used for this calculation. All correlations were statistically significant (p < 0.0001) and in the expected directions (Table 3).

### Item reduction

The item *Vomiting and Nausea *was suggested to be removed by one of the expert panel members. This item was according to the scale score distribution, beyond the limits set in this study. However, this item should remain, since *Vomiting and Nausea *is common in patients with IBS [39], especially among women [33]. In the present 37 percent of the women scored 50 or less on this item, indicating that symptoms were present.

Four of the expert panel members suggested that the item *Overall bowel symptoms *could be excluded, which was also the intension, since the item was used only as a control item. There were correlations between the item *Overall bowel symptoms *and the items concerning the individual's bowel symptoms; *Abdominal Pain *(*r *= 0.659, *p *< 0.0001), *Diarrhoea *(*r *= 0.286, *p *< 0.034), *Bloating and Flatulence *(*r *= 0.457, *p *< 0.0001), and *Vomiting and Nausea *(*r *= 0.284, *p *< 0.036). However, there was no correlation between the overall question and the item concerning *Constipation *(*r *= 0.232, *p *< 0.089). There was no correlation above 0.80, as required limit for removal, between any of the items in the VAS-IBS. However, there was a strong correlation between the item *Well-being during previous week *and the item *Perception of quality of life *(*r *= 0.762, *p *< 0.0001), and two experts also suggested that one of these items could be removed. To avoid misunderstanding of the sentence of the words "wellbeing" and "quality of life", these two items were replaced with the item *"How much/little have your gastrointestinal problems influenced your daily life?"*.

The internal validity in the VAS-IBS was high, since all items in the VAS-IBS questionnaires, which were handed in, were completed, and no data were missing. In total four items in the GSRS and the PGWB were left unanswered and they were replaced by the mean value of the dimension.

### Test-retest reliability

The test-retest reliability of the first version of the VAS-IBS had intra-class correlation coefficients ranging from 0.40 (*Vomiting and Nausea*) to 0.80 (*Constipation*). The test-retest showed acceptable values above 0.70 for each item except for *Vomiting and Nausea *and *Bloating and Flatulence *(0.63). There were no significant differences in scores between the first and second administrations.

### Acceptability of the scale

Agreement was achieved in the expert panel concerning the choice of the VAS as a preferable scale (Table 2). All items in the final version of the VAS-IBS except one, the item concerning *Daily life *(0–80), spanned the full range of the scale (0–100). The mean scores were situated near the midpoint of the scale (40–60) for all items except two namely; the item concerning *Bloating and Flatulence *(33.3), and the item concerning *Vomiting and Nausea *(66.1) (Table 4). The items' floor effects were low and ranged from 2.8 to 18.8 %. The ceiling effects range from 0 to 25.4 %, but only the item concerning *Vomiting and Nausea *was above the limit of 20 % set for this study (Table 4). All scale scores were in the acceptable range for skewness (Table 4).

### Internal consistency reliability

The psychometric testing of the final version of the VAS-IBS, based on 16 questionnaires, confirmed that the VAS-IBS is an acceptable, homogeneous, patient-reported questionnaire with internal consistency reliability. The overall VAS-IBS showed a high degree of internal consistency reliability as indicated by Cronbach's alpha internal consistency reliability coefficient, with a value of 0.85 (>0.70 required). As can be seen in Table 5 all the seven items performed well together as a composite measure. Each of the items had a high alpha value (0.81–0.85) if item was deleted.

### Other aspects

It took only a few minutes to calculate the scores in each VAS-IBS questionnaire and no manual was needed. The scores of each line were measured by a hand-held ruler. A conclusion based on the scores was easily reached and just by looking at the marks on the line, made by the patient, the health care professional could form an opinion of the patient's main complaints. The patients thought it was easy to complete the VAS-IBS and they were pleased that the questionnaire did not include more items.

## Discussion

The psychometric testing confirms that the VAS-IBS is an acceptable, homogeneous, patient-reported questionnaire with content and criterion validity and internal consistency reliability. The items in the VAS-IBS capture the main physical concerns women with IBS might present, although the expert panel suggested some changes. According to one panel member items related to *sensation of incomplete evacuation *and to *urgency to defecation*, could be added to the questionnaire. *Sensation of incomplete evacuation *was included in the first criterion for IBS [46], but was later transferred to the list of symptoms that cumulatively support the diagnosis of IBS [46]. After careful consideration, items on these issues could be added to the VAS-IBS as "yes" or "no" questions as a reminder of issues which should be discussed at the consultation. The result of these dialogues should be added to the patients' medical record. One panel member suggested adding an item about *food intolerance*, which is a good point, but the purpose of the VAS-IBS is to evaluate symptoms and not to discover reasons for the patients' symptoms. *Food intolerance *is a separate and complicated issue, and not a part of IBS. The item *Vomiting and Nausea *was suggested to be removed by one of the expert panel members, but should remain, since *Vomiting and Nausea *is common in patients with IBS [39], especially among women [10,33]. This item was according to the scale score distribution, beyond the limit for ceiling effect set in this study. However, since not all patients having IBS suffers from this symptom, this is an expected result.

In the present study, the time window for each item was set to the previous week in order to correlate with the time window in the GSRS and the PGWB. However, a longer period might be preferable to use in clinical practice, since it sometimes takes time before an improvement of a treatment can be achieved. As a suggestion, one month might be preferable, but it depends on the circumstances under which the VAS-IBS is to be used. Further testing of different time intervals is needed.

To choose VAS as the scaling method for the scoring of the items seems to be a correct choice. The items discriminated well between individuals according to the scale score distribution, and the mean agreement of the expert panel concerning the scale was also high. However, the items have not been weighted against each other, since the purpose was to create a questionnaire to follow each of the patient's complaints over time, and the individual items in the VAS-IBS should not be combined into sub-scales of each concept or be calculated into a total score.

The patients participating did not report any difficulties when using the VAS, although such problems have been reported [48]. The simplicity of using the questionnaire is important to maintain a high adherence among patients as well as among health care professionals. The questionnaire should not be a burden for them, but of assistance. To clarify for the patients, faces showing variations in their expression of symptoms might be added at the ends of the lines [49]. The purpose was to create a questionnaire to be used by all health care professionals to compare the outcome of different kinds of therapies in patients with established IBS according to current guidelines [3,6]. It is a strength that different health care professionals can use the VAS-IBS to assess the patients' status over time, but it might be a weakness that the questionnaire do not include the diagnostic criteria for IBS. Items concerning this issue were deliberately left out, since in Sweden only physicians are allowed to establish the diagnosis. The VAS-IBS should serve as a complement to the anamnesis and measure the response to treatment of symptoms related to IBS. However, symptoms, as for example diarrhoea and constipation, mean different things to different people. This fact may have been affected the statistical result concerning reliability, but in clinical practice this matter should not be a problem, since patients always will be compared to themselves over time.

There are limitations of this study and the stability of the VAS-IBS is one of them. The intra-class correlation coffecient for Bloating and Flatulence as well as for Vomiting and Nausea was low in the first version of the VAS-IBS. Patients' symptoms vary over time [47], especially bloating and nausea, and the results could be caused by the long time interval (four weeks) for the test-retest, so a shorter time window for that purpose had been preferable. The final version of the VAS-IBS needs to be further tested by assessing the sensitivity to change in use in clinical practice.

The capacity of the VAS-IBS to respond to treatments has not been judged. It is a challenge to establish cut-off points between statistical changes and those changes which benefit the patient, and one of the major problems is the lack of a definition of what should be accepted as a clinical improvement of the patients' complaints. Patients suffering from IBS are a heterogeneous group, and no biological makers are available to quantify the severity of IBS or a change in severity, which makes the judgments more difficult. However, a small degree on VAS could be clinically meaningless, but this problem can appear in all kinds of scales methods [15].

Also the selection of the patients in this study ought to be discussed. It was difficult to recruit patients to take part in the study, partly due to the treatment they had been given during the years of their illness [50]. If the participants had been taken better care of, they would also have been more eager to play a part in the research projects. The high number of patients' not participating in this study may be a limitation. We only know the diagnosis, age and the reason for refraining concerning the patients not included. It is likely that the women who took part in the study were especially interested, since all patients included, who were asked to complete a questionnaire, fulfilled it. The VAS-IBS has only been used for research and tested only on Swedish women suffering from IBS, and needs therefore to be used in clinical practise, at primary as well as at secondary care level, cross-culturally, in women as well as in men before its clinical significance can be established. Before the VAS-IBS can be used in other languishes than Swedish, the VAS-IBS needs to be properly translated and validated in the actual languish.

Parallel to the present study the GSRS-IBS was developed [51] and evaluated on patients in the USA and the UK. The idea was to construct a questionnaire which could be used in clinical research. The GSRS-IBS is a short, user-friendly, 13-item questionnaire and a seven-point Likert scale is used for each item. In the GSRS-IBS items *sensation of incomplete evacuation *and *urgency to defecation *are included, but items concerning *vomiting and/or nausea *are missing. Since the VAS-IBS and the GSRS-IBS include different items and use different scales, they could both be useful in clinical practice. However, the VAS-IBS might be preferable, since it has fewer items and requires few calculations.

## Conclusion

The items in the VAS-IBS capture the main physical concerns women with IBS might exhibit. The VAS-IBS is designed to detect differences in five main complaints related to bowel symptoms, mental health as well as daily life, and is intended to be used in clinical practice as a complement to the anamnesis. The VAS-IBS seems to be a valid and reliable questionnaire and appears to be user-friendly, for patients as well as for health professionals. The questionnaire is easy to complete and unproblematic to calculate. It is possible to quickly form an opinion of the patients' complaints. As a result of the psychometric testing the VAS-IBS could serve as a useful questionnaire in different situations to quantify the impact that gastrointestinal symptoms may have on patients with IBS. However, the VAS-IBS has to be used in clinical practice and further tested before the clinical significance can be established. The time window is set to one month in the final version of the VAS-IBS (Appendix 1), but further testing of different time intervals is needed.

## Competing interests

The authors declare that they have no competing interests in relation to this manuscript. The principal author (MB) received financial support in the form of study grant and postgraduate studentship from the Faculty of medicine, Lund University, Sweden, but there are non-financial or financial competing interests to declare.

## Authors' contributions

MB has substantial contributions to conception and design, acquisition of data, analysis and interpretion of data and mainly drafting the manuscript. BO has been involved in revising it critically for important intellectual content. KU has contributed with analysis and interpretion of data and has been involved in revising the manucript critically. All authors read and approved the final manuscript.

## Appendix 1

Visual Analogue Scale for Irritable Bowel Syndrome (VAS-IBS)

How disturbing during the last month have your problems concerning your:

abdominal pain ?

diarrhoea?

constipation?

bloating and flatulence?

vomiting and nausea?

How do you rate your mental well-being over the past month?

How much/little have your gastrointestinal problems influenced your daily life over the past month?

Have you during the last month felt urgency to defecation? YES/NO

Have you during the last month felt that your bowel has not

been completely empty after visiting the toilette? YES/NO

Bengtsson et al.

## Pre-publication history

The pre-publication history for this paper can be accessed here:



## Figures and Tables

**Table 1:** The criteria for the diagnosis Irritable Bowel Syndrome according to the Rome II and III consensus

*Rome II: *At least 12 weeks, which need not be consecutive, in the preceding 12 months, of abdominal discomfort or pain that has two of three features:
*Rome III: *At least 3 months, with onset at least 6 months previously of recurrent abdominal pain or discomfort associated with 2 or more of the following:

(1) Relieved with defecation; and/or
(2) Onset associated with a change in frequency of stool; and/or
(3) Onset associated with a change in form (appearance) of stool.

Supportive symptoms:
1. Fewer than 3 bowel movements a week
2. More than 3 bowel movements a day
3. Hard or lumpy stools
4. Loose (mushy) or watery stools
5. Straining during a bowel movement
6. Urgency (having to rush to have a bowel movement)
7. Feeling of incomplete bowel movement
8. Passing mucus (white material) during a bowel movement
9. Abdominal fullness, bloating or swelling

(Rome II: Thompson et al. 1999; p1144, Rome III: Chang 2006).

**Table 2:** The expert panel members' (n = 9) agreement on the items in the Visual Analogue Scale for Irritable Bowel Syndrome (VAS-IBS) concerning content validity (relevance and definition) and acceptability of the scale.

	**Relevance**		**Definition**		**Acceptability of the scale**	
	Score 4 and 3	Score 2 and 1	Score 4 and 3	Score 2 and 1	Score 4 and 3	Score 2 and 1
**Physical symptoms**						
Overall item bowel symptoms	8 (6+2)	1	8 (4+4)	1	9 (8+1)	0
Abdominal Pain	9 (8+1)	0	9 (6+3)	0	8 (6+2)	1
Diarrhoea	9 (8+1)	0	8 (6+2)	1	8 (6+2)	1
Constipation	9 (8+1)	0	8 (6+2)	1	8 (6+2)	1
Bloating and Flatulence	8 (7+1)	1	8 (6+2)	1	9 (7+2)	0
Vomiting and Nausea	8 (6+2)	1	8 (5+3)	1	8 (7+1)	1
**Mental health**						
Perception of mental well-being	9 (6+3)	0	9 (6+3)	0	9 (9+0)	0
**Quality of Life**						
Well-being previous week	9 (8+1)	0	9 (6+3)	0	9 (9+0)	0
Perception of quality of life	9 (9+0)	0	9 (8+1)	0	9 (9+0)	0

The level of agreement was set to no more than one panel member scoring an item at less than 3.

**Table 3:** Criterion validity (Pearson's correlation test) between the Visual Analogue Scale for Irritable Bowel Syndrome (VAS-IBS) and the Gastrointestinal Symptom Rating Scale (GSRS) as well as the Psychological General Well Being Index (PGWB) (n = 71).

**VAS-IBS**	**GSRS- Pain**	**GSRS- Diarrhoea**	**GSRS- Constipation**	**GSRS- Indigestion**	**GSRS- item Vomiting/nausea**	**PGWB**
**Physical symptom**						
Abdominal Pain	-0.597					
Diarrhoea		-0.806				
Constipation			-0.770			
Bloating and Flatulence				-0.664		
Vomiting and Nausea					-0.814	
**Mental health**						
Perception of mental well-being						0.689

Values are given as correlation coefficients and are all statistically significant, p < 0.0001.

**Table 4:** The score range, mean as well as floor and ceiling effects and skewness of each item in the final version of the Visual Analogue Scale for Irritable Bowel Syndrome (VAS-IBS), as scored by the participating women (n = 71).

**VAS-IBS**	Score Range*	**Mean (SD)**	**Floor effects %**	**Ceiling effects %**	**Skewness**
**Physical symptoms**					
Abdominal Pain	0–100	46.5 (29.4)	4.2	7.0	0.444
Diarrhoea	0–100	60.0 (34.3)	7.1	20.0	-0.283
Constipation	0–100	58.8 (32.9)	2.8	12.7	-0.226
Bloating and Flatulence	0–100	33.3 (27.1)	8.5	1.4	0.774
Vomiting and Nausea	0–100	66.1 (33.8)	4.2	25.4	-0.543
**Mental health**					
Perception of mental well-being	0–100	53.2 (30.0)	5.5	5.6	-0.044
**Quality of Life**					
Influence daily life **	0–80	43.6 (29.2)	18.8	0	-0.297

The limit of the floor and ceiling effect was set at 20% or less and the skewness should be between -1 and +1 to be acceptable. 0 = worst symptom/health and 100 = best symptom/health

** n = 16

**Table 5:** The internal consistency of the final version of the Visual Analogue Scale for Irritable Bowel Syndrome (VAS-IBS) (Cronbach's alpha) (n = 16).

**VAS-IBS**	**Alpha-if-item deleted**	**Cronbach's alpha coefficient**
**Physical symptoms**		
Abdominal Pain	0.83	
Diarrhoea	0.85	
Constipation	0.85	
Bloating and Flatulence	0.83	
Vomiting and Nausea	0.81	
**Mental health**		
Perception of mental well-being	0.81	
**Quality of Life**		
Interference with daily life	0.83	
**Overall scale**		0.85

A minimum coefficient of 0.70 was required
